# Effects of Dynamic IMU-to-Segment Misalignment Error on 3-DOF Knee Angle Estimation in Walking and Running

**DOI:** 10.3390/s22229009

**Published:** 2022-11-21

**Authors:** Chao Jiang, Yan Yang, Huayun Mao, Dewei Yang, Wei Wang

**Affiliations:** 1 Biomedical Engineering Research Center, School of Bioinformatics, Chongqing University of Posts and Telecommunications, No. 2 Chongwen Road, Chongqing 400065, China; 2 School of Automation, Chongqing University of Posts and Telecommunications, No. 2 Chongwen Road, Chongqing 400065, China; 3 School of Advanced Manufacturing Engineering, Chongqing University of Posts and Telecommunications, No. 2 Chongwen Road, Chongqing 400065, China

**Keywords:** IMU, DPSO, joint constraint, dynamic IMU-to-segment alignment

## Abstract

The inertial measurement unit (IMU)-to-segment (I2S) alignment is an important part of IMU-based joint angle estimation, and the accurate estimation of the three degree of freedom (3-DOF) knee angle can provide practical support for the evaluation of motions. In this paper, we introduce a dynamic weight particle swarm optimization (DPSO) algorithm with crossover factor based on the joint constraint to obtain the dynamic alignment vectors of I2S, and use them to perform the quaternion-based 3-DOF knee angle estimation algorithm. The optimization algorithm and the joint angle estimation algorithm were evaluated by comparing with the optical motion capture system. The range of 3-DOF knee angle root mean square errors (RMSEs) is 1.6°–5.9° during different motions. Furthermore, we also set up experiments of human walking (3 km/h), jogging (6 km/h) and ordinary running (9 km/h) to investigate the effects of dynamic I2S misalignment errors on 3-DOF knee angle estimation during different motions by artificially adding errors to I2S alignment parameters. The results showed differences in the effects of I2S misalignment errors on the estimation of knee abduction, internal rotation and flexion, which indicate the differences in knee joint kinematics among different motions. The IMU to thigh misalignment error has the greatest effect on the estimation of knee internal rotation. The effect of IMU to thigh misalignment error on the estimation of knee abduction angle becomes smaller and then larger during the two processes of switching from walking to jogging and then speeding up to ordinary running. The effect of IMU to shank misalignment error on the estimation of knee flexion angle is numerically the largest, while the standard deviation (SD) is the smallest. This study can provide support for future research on the accuracy of 3-DOF knee angle estimation during different motions.

## 1. Introduction

Walking and running are the most common human motions in daily life, and the knee plays an important role in these motions, due to the constant repetition of similar movements, most motion injuries occur in the lower limb’s knee [1]. Accurate gait analysis before lesions occur can provide non-surgical behavior modification treatment for osteoarthritis patients, reducing the damage to their joints [2]. Consequently, a growing number of gait analysis techniques are providing diagnostic support to clinicians. The accuracy of the joint angle estimation determines the possibility of implementing this technique.

Compared to infrared-based optical motion capture, inertial measurement unit (IMU)-based gait analysis is increasingly studied and applied due to its superior portability and good accuracy [3,4]. The IMU-based joint angle estimation mainly involves the conversion between global coordinates, IMU coordinates and segment coordinates [5]. The IMU-to-segment (I2S) alignment determines whether the data output from the IMUs is representative of the movement of the attached segment [6,7,8].

There are four general approaches to the I2S alignment [5].

(1) The first approach is that the professionals attach IMUs to the subject according to the bone coordinates recommended by the international society of biomechanics (ISB) [9] to do the manual alignment. A validation of this approach in knee flexion and extension is provided in study [10]. Obviously, this approach cannot eliminate the errors brought by the movement process, and requires the presence of professional IMU placement personnel for each acquisition, which makes this approach unsuitable for universal applications.

(2) The second approach is to ask subjects to perform a series of pre-actions before the experiment, and then to conduct I2S alignment by processing this pre-action information. In study [11], subjects are required to complete a sequence of pre-actions to identify joints, and the authors also pointed out that defining anatomical frameworks can be only repeated within subjects, but across subjects. Although the pre-actions can eliminate the errors of the IMUs setting, they cannot eliminate the errors caused by the relative displacement between the IMUs and the segments during the movement. Moreover, it is difficult to judge whether the subjects’ pre-actions are standard, which brings challenges to practical applications.

(3) The third approach is to introduce information from other equipment to do the I2S alignment. In study [12], researchers proposed a lower extremity surgery approach based on an additional optical motion capture anatomical framework. The impracticality of the extra equipment makes this approach less studied.

(4) The fourth approach is to use some mathematical constraint model to conduct the I2S alignment. The joint constraint proposed by the study [13] is to optimize the data output of the IMUs by using the mathematical constraint model of the joint during movements, then the data output by the IMUs can more accurately represent the movement of the segment. In this study, we chose this approach to obtain the dynamic I2S alignment parameters because this approach has no limitations on movements, and there is no requirement for IMU placement or pre-actions, which reduces artificial errors and can be easily applied.

About the joint angle estimation, many studies have focused on analyzing the accuracy of joint angle estimation algorithms [14,15], but the exact effect of errors on the results remains unclear. In study [16], the effect of anatomical frame variation on joint angles was investigated by an optical motion capture system, the high demands of the experimental environment make it difficult to be a universal method. In study [17], the effects of static I2S misalignment error and IMU rotation error on 3-DOF knee angle estimation during drop landing and cutting tasks were investigated, it was shown that the abduction angle and internal rotation angle of the knee were more sensitive to I2S misalignment error, while the flexion angle was more sensitive to IMU rotation error, but it is difficult to evaluate the effect of static I2S alignment parameters on the motion process.

The significant differences in the kinematics and kinetics of the knee joint during walking and running at different speeds have been confirmed by many studies [18]. The peak of knee flexion was higher in walking than in running due to greater knee extension [19]. Regardless of whether the subject is healthy or not, a large number of kinematic parameters of the subject are changed as a result of the speed-up [20]. In the study [21], a nonlinear transition in the 6-DOF kinematics of the knee was found not only for velocity changes in the same motion but also for different motions, such as switching from walking to running. This means that it is worthwhile to perform an accurate estimation of the knee angle in different motions.

The main purpose of this paper is to investigate the effects of dynamic I2S misalignment error on 3-DOF knee angle estimation in walking and different speeds of running. Motion data from the thigh and shank are collected using IMUs and optical motion capture systems while walking (3 km/h), jogging (6 km/h) and running (9 km/h). We introduce the dynamic weight particle swarm optimization (DPSO) algorithm with crossover factor to solve the joint constraint, obtain the dynamic I2S alignment parameters and finally perform the estimation of 3-DOF knee angle based on quaternions. Furthermore, the specific effect of dynamic I2S misalignment errors on 3DOF knee angle estimation is investigated by adding manual errors to the I2S alignment parameters. Through experiments, the performance of the optimization algorithm and joint angle estimation algorithm is evaluated, the exact effects of misalignment error on 3-DOF knee angle estimation in different motions were studied.

## 2. Methods and Equipment

### 2.1. Methods

#### 2.1.1. IMU-to-Segment Alignment Based on Joint Constraints

(1) Joint Constraint

**Figure 1:**
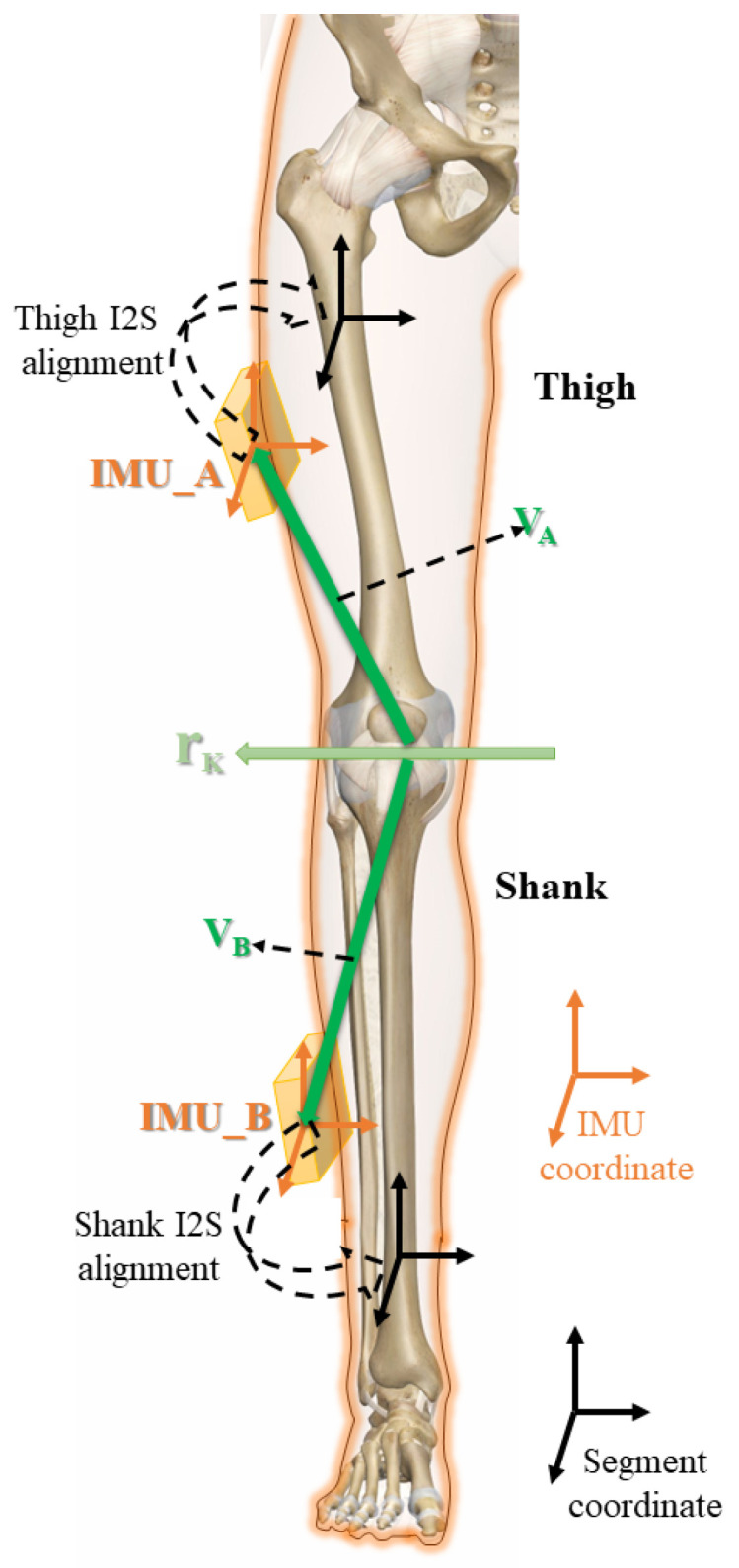
Illustrations of joint constraints and I2S alignment. The schematic diagram of each vector in the joint constraint model is shown with colored arrows; the coordinates of IMU and segment are shown with different colors; the black dashed arrows indicate the alignment of IMU to segment.

For the spherical and hinge joints, as shown in Figure 1, the coordinates of the limb segments are in black [9], the brown cubes attached to the segments represent the IMUs, and the coordinates of them are represented in brown. The acceleration and angular velocity data collected by the IMUs attached to the two segments meet different constraints [13].

For the hinge joint, the unit vector $r_{k}$ parallel to the joint axis shown in Figure 1 is defined in the coordinates of the two IMUs A,B denoted as $r_{k,A}$, $r_{k,B}$, respectively. Under ideal conditions, the angular velocity information $\omega_{i},i\in \left\{A,B\right\}$ obtained from IMUs satisfies the constraint:

(1) $$\|\omega_{A}\left(t\right)\times r_{k,A}\left\|-\right\|\omega_{B}\left(t\right)\times r_{k,B}\|=0$$

where $∥\cdot ∥$ denotes the modulus of a vector and × denotes the cross product of vectors. Converting $r_{k,A}$, $r_{k,B}$ from Cartesian to spherical coordinates:

(2) $$r_{k,i}=\left[\cos\varphi_{i}\cos\theta_{i},\cos\varphi_{i}\sin\theta_{i},\sin\theta_{i}\right]\;,i\in \left\{A,B\right\}$$

where the pitch angle $\varphi \in \left[0,\pi \right]$ and the yaw angle $\theta \in \left[0,2\pi \right]$, and the error $e_{J}\left(t\right)\left(\varphi_{A},\varphi_{B},\theta_{A},\theta_{B}\right)$ is abbreviated as $e_{J}\left(t\right)$. Since the IMU coordinates are not aligned with the segment coordinates, the IMU data do not exactly satisfy Equation (1), and we need to optimize the data, then the error function is expressed as:

(3) $$e_{J}\left(t\right)=\|\omega_{A}\left(t\right)\times r_{k,A}\left\|-\right\|\omega_{B}\left(t\right)\times r_{k,B}\|$$

For spherical joints, define vectors $V_{A}$ and $V_{B}$ pointing from the center of the sphere to the origin of IMU coordinates, expressed in IMUs coordinates as:

(4) $$V_{i}=\left[X_{i},Y_{i},Z_{i}\right]\;,i\in \left\{A,B\right\}$$

Under ideal conditions, the acceleration $a_{i},i\in \left\{A,B\right\}$ and angular velocity $\omega_{i},i\in \left\{A,B\right\}$ obtained from IMUs satisfy the constraint:

(5) $$\begin{array}{cc} ∥a_{A}\left(t\right)-\Gamma_{A}\left(t\right)∥- & ∥a_{B}\left(t\right)-\Gamma_{B}\left(t\right)∥=0,\\ \Gamma_{i}\left(t\right)=\omega_{i}\left(t\right)\times (\omega_{i}\left(t\right)\times & V_{i})+\alpha_{i}\left(t\right)\times V_{i}\;,i\in \left\{A,B\right\} \end{array}$$

where $\alpha_{i}\left(t\right)$ denotes the angular acceleration calculated from the angular velocity measured by IMUs, we choose the third-order approximation to calculate the time derivative of the angular velocity $\omega_{i}$:

(6) $$\alpha_{i}=\frac{\omega_{i}\left(t-2▵t\right)-8\omega_{i}\left(t-▵t\right)+8\omega_{i}\left(t+▵t\right)-\omega_{i}\left(t+2▵t\right)}{12▵t}\;,i\in \left\{A,B\right\}$$

Since the IMU coordinates are not aligned with the segment coordinates, the IMU data do not exactly satisfy Equation (5), and we need to optimize the data. The error $e_{S}\left(t\right)\left(X_{A},Y_{A},Z_{A},X_{B},Y_{B},Z_{B}\right)$ is abbreviated as $e_{S}\left(t\right)$, then the error function is expressed as:

(7) $$e_{S}\left(t\right)=∥a_{A}\left(t\right)-\Gamma_{A}\left(t\right)∥-∥a_{B}\left(t\right)-\Gamma_{B}\left(t\right)∥$$

(2) Optimization Algorithm

The above problems can be solved by various optimization algorithms. In study [22], the author uses Gauss–Newton (GN) to solve the optimization problem of joint constraints, but a new mechanism must be introduced to avoid the final optimization results $r_{k,A}$ and $r_{k,B}$ of $e_{J}\left(t\right)$ produce an unrealistically negative value. Moreover, the calculation of a large number of Jacobian matrices and Hessian matrices made the algorithm have a very unsatisfactory calculation speed.

In this study, due to the non-convexity of the error function, the classical PSO algorithm can make the optimization results fall into a local optimum. Study [23] showed that the meta-heuristic DPSO and gray wolf optimization algorithm (GWO) have better accuracy than GN, and the advantage is more obvious when joint rotation is not obvious. Inspired by the genetic algorithm (GA) with gene crossover variation, we introduced the DPSO algorithm with crossover factor.

The introduced algorithm avoids the extremely complicated coding and decoding steps of GA, does not need to calculate a large number of Jacobi matrices and Hesse matrices, and the initial range of particles set in the positive range can also avoid the problem of unrealistic negative values of GN’s optimization results.

Joint constraints for hinge and spherical joints can be considered as four-dimensional and six-dimensional search optimization problems. The basic particle swarm optimization is a set of particles moving in the search space, the best position of a single particle at past time is pbest and the best position for all particles is gbest.

The velocity update formula of particle n as Equation (8):

(8) $$\begin{array}{cc} V_{v,n}^{k}= & w*V_{v,n}^{k-1}+c_{1}*rand_{1}*\left({\mathrm{pbest}}_{n}-V_{n}^{k-1}\right)\\ \; & +c_{2}*rand_{2}*\left({\mathrm{gbest}}_{n}-V_{n}^{k-1}\right) \end{array}$$

The position update formula of particle n as Equation (9):

(9) $$V_{n}^{k}=V_{n}^{k-1}+V_{v,n}^{k}$$

where $V_{v,n}^{k}$ represents the velocity vector of the *k-th* iteration of *n* particles. $V_{n}^{k}$ represents the position vector of the *k-th* iteration of the *n* particle, i.e., $V_{n}^{k}=\left(X_{A},Y_{A},Z_{A},X_{B},Y_{B},Z_{B}\right)$; $c_{1}$, $c_{2}$ represent acceleration constants, which can adjust the maximum step size of learning; $rand_{1}$ and $rand_{2}$ are two random functions with a range of [0,1], which can make the search more random; *w* represents the inertia weight of non-negative number, which adjusts the search range of the space. In order to ensure the large-scale search in the early iterations and the convergence, in this study, the value of *w* is determined as:

(10) $$w=w_{max}-\frac{T*(w_{max}-w_{min})}{T_{max}}$$

where $w_{max}$ and $w_{min}$ are the upper and lower bounds of the inertia weight, *T* is the current iterations of the particle, and $T_{max}$ is the maximum iterations of the particle. It can be seen from the Equation (10) that the particle will search with a large step in the early iterations, as the number of iterations increases, the step size becomes smaller.

**Figure 2:**
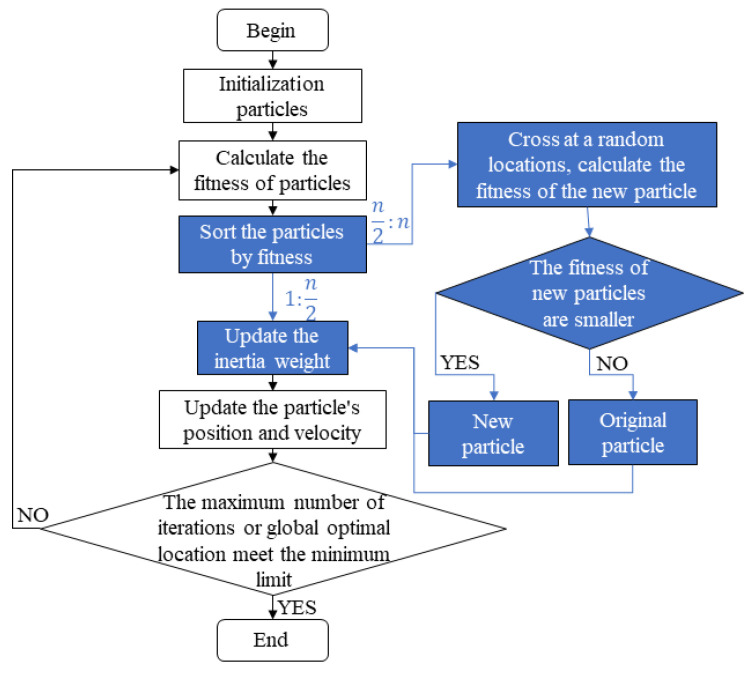
Flow chart of introduced optimization algorithm.

Figure 2 shows the flowchart of the introduced algorithm. Compared with the original particle swarm optimization, the blue part was added, including the dynamic inertia weight *w* and the sorting of n particles according to their fitness from small to large. After sorting, the first half of the $1:\frac{n}{2}$ particles are used for normal particle swarm iteration, and the other half of the $\frac{n}{2}:n$ particles are selected for crossover. The specific steps are:

(1) Choose a section of the particle at random;

(2) Randomly exchange elements of this section between particles to obtain new particles;

(3) Calculate the fitness of the new particle, compare it with the original particle;

(4) Retain particles with lower fitness for the next iteration of the particle swarm.

#### 2.1.2. Quaternion-Based 3-DOF Knee Angle Estimation

Establish the coordinates attached to limbs [23], combining the results of the optimization algorithm in the previous section to obtain $V_{A},V_{B},r_{k,A},r_{k,B}$, we can obtain the rotation matrix $R_{A}^{Thigh},R_{B}^{Shank}$ of the I2S alignment:

(11) $$\begin{array}{cc} R_{A}^{Thigh}= & {\left[\frac{V_{A}}{∥V_{A}∥}\times r_{k,A},\;\frac{V_{A}}{∥V_{A}∥},\;r_{k,A}\right]}^{T},\\ R_{B}^{Shank}= & {\left[\frac{V_{B}}{∥V_{B}∥}\times r_{k,B},\;\frac{V_{B}}{∥V_{B}∥},\;r_{k,B}\right]}^{T} \end{array}$$

where $R_{A}^{Thigh}$ denotes the rotation matrix from IMU_A to the thigh and $R_{B}^{Shank}$ denotes the rotation matrix from IMU_B to the shank. The elements of the rotation matrix are not independent, and it is difficult to add Euler angular errors, which are tedious to calculate and can be subsequently calculated by converting them into quaternions.

The general method for converting a rotation matrix to a quaternion is that we have the known rotation matrix R:

(12) $$R=\left[\begin{array}{ccc} m_{00} & m_{01} & m_{02}\\ m_{10} & m_{11} & m_{12}\\ m_{20} & m_{21} & m_{22} \end{array}\right]$$

convert the elements of the matrix:

(13) $$\begin{array}{cc} q_{w}= & \frac{1}{2}\sqrt{tr(R)}\\ q_{x}= & \frac{m_{21}-m_{12}}{4q_{w}}\\ q_{y}= & \frac{m_{02}-m_{20}}{4q_{w}}\\ q_{z}= & \frac{m_{10}-m_{01}}{4q_{w}} \end{array}$$

where tr(R) is the trace of the matrix, the converted unit quaternion q can be expressed as:

(14) $$q=\left[q_{w},q_{x},q_{y},q_{z}\right]$$

when the absolute value of $q_{w}$ is small, it will cause the converted value to be unstable, so we need to compare the values of $q_{w},q_{x},q_{y},q_{z}$ before outputting the quaternion [24]. Therefore, we obtain the dynamic I2S alignment quaternion $q_{T,ali}$, $q_{S,ali}$.

The thigh and shank orientation, $q_{T},q_{S}$, can be calculated according to IMU orientation quaternion and the alignment quaternion, as in:

(15) $$\begin{array}{cc} q_{T}= & q_{A}⊗q_{T,ali}^{*}\\ q_{S}= & q_{B}⊗q_{S,ali}^{*} \end{array}$$

where $q_{A}$ and $q_{B}$ are the orientation quaternions of IMU_A and IMU_B, $q_{T,ali}^{*}$ and $q_{S,ali}^{*}$ denote the conjugate quaternions of $q_{T,ali}$ and $q_{S,ali}$.

Through the thigh orientation $q_{T}$ and shank orientation $q_{S}$, the knee angle $q_{TS}$ can be calculated as the orientation of shank relative to thigh, as in:

(16) $$q_{TS}=q_{T}^{*}⊗q_{S}$$

Supposing that $q_{TS}=\left[\omega ,x,y,z\right]$, the estimated knee angle is the ‘ZYX’ sequence Euler angle representation of $q_{TS}$, and the X, Y and Z axes correspond to the axes of knee abduction (AA), knee internal rotation (IE) and knee flexion (FE), which means the 3-DOF knee angle can be described as follows:

(17) $$\begin{array}{cc} \; & \phi_{AA}=atan2\left(\left(2*\left(\omega *x+y*z\right)\right),\left(1-2*\left(x^{2}+y^{2}\right)\right)\right)\\ \; & \theta_{IE}=asin\left(2*\left(\omega *y-x*z\right)\right)\\ \; & \psi_{FE}=atan2\left(\left(2*\left(\omega *z+y*x\right)\right),\left(1-2*\left(y^{2}+z^{2}\right)\right)\right) \end{array}$$

Similarly, the 3-DOF knee angle can be calculated based on the segment rotation data from the optical motion capture system $\psi_{FE\_opt},\theta_{IE\_opt},\phi_{AA\_opt}$, as reference.

#### 2.1.3. Adding Errors to the Dynamic I2S Alignment Parameter

To analyze the effects of dynamic I2S misalignment errors on the estimation of 3-DOF knee angles during walking, jogging and ordinary running, we need to manually add errors to the alignment quaternion $q_{T,ali}$ and $q_{S,ali}$. It is not intuitive to add errors directly to the quaternion, we need to convert the alignment quaternion to Euler angular roll, pitch and yaw.

By Equation (17), the alignment quaternion can be converted into Euler angles $\psi_{ali},\theta_{ali},\phi_{ali}$, add the error Euler angles $\psi_{er},\theta_{er},\phi_{er}$ from −10° to +10° in steps of 5° to them, respectively, obtain the new alignment Euler angles $\psi_{ali\_er},\theta_{ali\_er},\phi_{ali\_er}$ as follows:

(18) $$\begin{array}{cc} \psi_{ali\_er}= & \psi_{ali}+\psi_{er}\\ \theta_{ali\_er}= & \theta_{ali}+\theta_{er}\\ \phi_{ali\_er}= & \phi_{ali}+\phi_{er} \end{array}$$

convert the new alignment Euler angles into alignment quaternions $q_{ali\_er}$:

(19) $$\begin{array}{cc} \; & q_{ali\_er}\left(\psi_{ali\_er},\theta_{ali\_er},\phi_{ali\_er}\right)=\\ \; & \left[\begin{array}{c} \cos\left(\frac{1}{2}\psi_{ali\_er}\right)*\cos\left(\frac{1}{2}\theta_{ali\_er}\right)*\cos\left(\frac{1}{2}\phi_{ali\_er}\right)+\sin\left(\frac{1}{2}\psi_{ali\_er}\right)*\sin\left(\frac{1}{2}\theta_{ali\_er}\right)*\sin\left(\frac{1}{2}\phi_{ali\_er}\right)\\ \cos\left(\frac{1}{2}\psi_{ali\_er}\right)*\cos\left(\frac{1}{2}\theta_{ali\_er}\right)*\sin\left(\frac{1}{2}\phi_{ali\_er}\right)-\sin\left(\frac{1}{2}\psi_{ali\_er}\right)*\sin\left(\frac{1}{2}\theta_{ali\_er}\right)*\cos\left(\frac{1}{2}\phi_{ali\_er}\right)\\ \sin\left(\frac{1}{2}\psi_{ali\_er}\right)*\cos\left(\frac{1}{2}\theta_{ali\_er}\right)*\sin\left(\frac{1}{2}\phi_{ali\_er}\right)+\cos\left(\frac{1}{2}\psi_{ali\_er}\right)*\sin\left(\frac{1}{2}\theta_{ali\_er}\right)*\cos\left(\frac{1}{2}\phi_{ali\_er}\right)\\ \sin\left(\frac{1}{2}\psi_{ali\_er}\right)*\cos\left(\frac{1}{2}\theta_{ali\_er}\right)*\cos\left(\frac{1}{2}\phi_{ali\_er}\right)-\cos\left(\frac{1}{2}\psi_{ali\_er}\right)*\sin\left(\frac{1}{2}\theta_{ali\_er}\right)*\sin\left(\frac{1}{2}\phi_{ali\_er}\right) \end{array}\right] \end{array}$$

According to Equations (15) and (16), the orientation of shank relative to thigh with error $q_{TS\_er}$ can be calculated:

(20) $$q_{TS\_er}={\left(q_{A}⊗q_{T,ali\_er}^{*}\right)}^{*}⊗\left(q_{B}⊗q_{S,ali\_er}^{*}\right)$$

As Equation (17), we can obtain the 3-DOF knee angle with manually added I2S misalignment errors: $\psi_{FE,Add\_er},\theta_{IE,Add\_er},\phi_{AA,Add\_er}$.

#### 2.1.4. Data Analysis

The standard deviation (SD) indicates the dispersion of the errors and can be calculated by the following equation:

(21) $$SD=\sqrt{\frac{\sum_{i=1}^{N}{\left(\beta_{i}-\mu_{\beta }\right)}^{2}}{N}}$$

where β denotes the error, $\mu_{\beta }$ denotes the mean of the error and *N* is the number of samples.

The root mean square error (RMSE) represents the deviation between the observed and reference values, and the square of the difference between them can increase the effect of larger errors, obtained through the following equation:

(22) $$RMSE=\sqrt{\frac{\sum_{i=1}^{N}{\left(X_{i}-x_{i}\right)}^{2}}{N}}$$

where $X_{i}$ denotes the observed value and $x_{i}$ denotes the reference value.

### 2.2. Measurement Equipment

The IMUs selected for this study were the wireless nine-axis inertial sensors Xsens Dot (±2000 deg/s, ±16 g) from Xsens (Netherlands).

Twelve high-speed infrared cameras from Nokov (Beijing, China) form an optical motion capture system to capture the position of markers, and both systems operate at 60 Hz.

**Figure 3:**
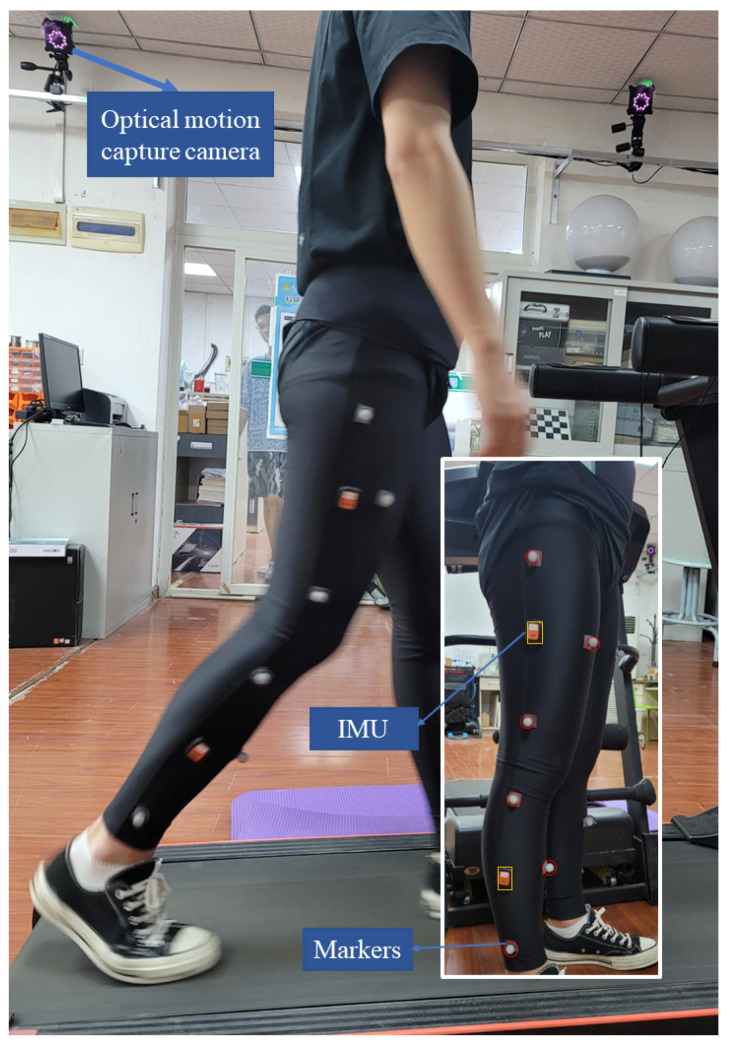
The experimental scenario mainly includes an optical motion capture system, IMUs and a treadmill; six markers and two IMUs are set on each leg, three markers on each segment are not co-planar and the direction of IMUs is not required.

Figure 3 shows that the IMUs are bound to the thigh and shank of both legs to measure the rotation data of the attached segment. The three non-coplanar markers are set on the thighs and shank of both legs to define the attached rigid bodies, and the rotational data of each segment is output through Nokov’s software.

### 2.3. Participants

Five healthy subjects (age 24 ± 2 years, height 175 ± 5 cm, mass 65 ± 5 kg) participated in this study. Every subject wearing IMUs and markers did walking, jogging and ordinary running experiments on a treadmill at speeds of 3 km/h, 6 km/h and 9 km/h, respectively, and each task was repeated three times. All subjects volunteered to participate in the experiment after being informed of the specific details and possible risks prior to participation.

## 3. Results

### 3.1. Joint Constraint Optimization

**Figure 4:**
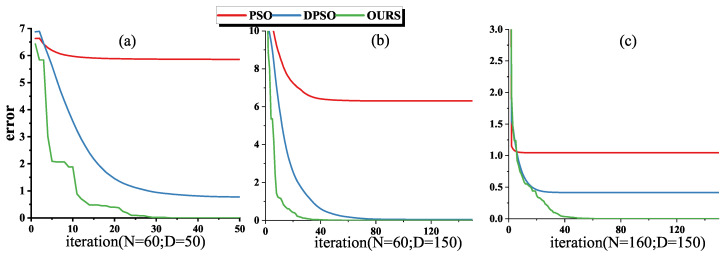
Convergence curves of three algorithms under different combinations of particle swarm size and iteration number, N denotes the particle swarm size and D denotes the maximum number of iterations, the red, blue and green lines represent the convergence curves of the classical PSO, DPSO and our introduced algorithm, respectively.

As in Figure 4, the collected knee (i.e., IMU_A and IMU_B) data were processed with PSO, DPSO and DPSO with crossover factor, respectively. Experiments were carried out at the particle swarm size N = 60, the maximum number of iterations D = 50; N = 60, D = 150 and N = 160, D = 150, respectively. The stability and accuracy of the algorithm are studied under various combinations of population size and iteration.

### 3.2. Knee Joint Angle Estimation Results

Similar to the knee angle estimation algorithm established in Section 2, we obtain three sets of data: 3-DOF knee angles based on IMU data after joint constraints $\psi_{FE},\theta_{IE},\phi_{AA}$; based on IMU data with artificially added error sequences in the I2S alignment parameter $\psi_{FE,Add\_er},\theta_{IE,Add\_er},\phi_{AA,Add\_er}$ and based on the optical motion capture system $\psi_{FE\_opt},\theta_{IE\_opt},\phi_{AA\_opt}$.

By comparing the data, we analyzed the effects of I2S misalignment error on 3-DOF knee angle estimation during walking, jogging and ordinary running, also evaluate our knee angle estimation algorithm by comparing the $\psi_{FE},\theta_{IE},\phi_{AA}$ between $\psi_{FE\_opt},\theta_{IE\_opt},$$\phi_{AA\_opt}$.

#### 3.2.1. The Effects of Dynamic I2S Misalignment Error in Different Motions

**Figure 5:**
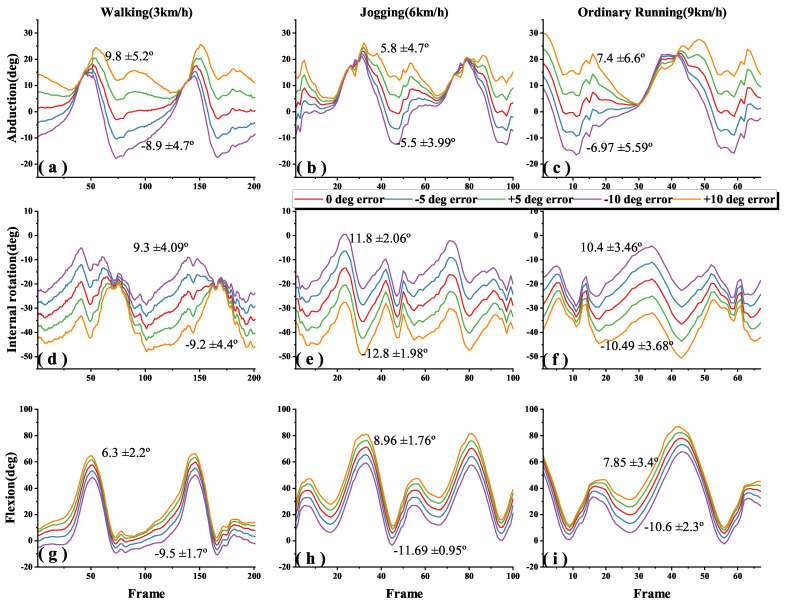
Effects of IMU to thigh misalignment error on 3-DOF knee angle estimation during different motions. The blue, green, purple and orange lines indicate the knee angle estimates after adding errors of −5°, +5°, −10° and +10° to the I2S alignment parameters, and the red line indicates the knee angle estimates without artificially added errors. (**a**–**c**), (**d**–**f**), (**g**–**i**) are the abduction, internal rotation and flexion angles of the knee during walking (3 km/h), jogging (6 km/h) and ordinary running (9 km/h) on treadmill. For example, in (**a**), 9.8 ± 5.2° denote that the abduction mean error is 9.8° and the SD is 5.2° when the introduced IMU to thigh misalignment error is +10° during the walking trial.

**Figure 6:**
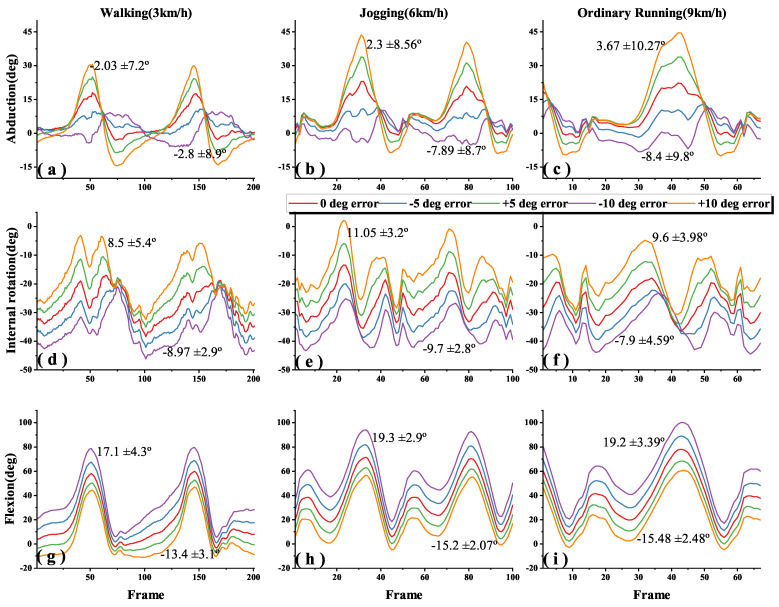
Effects of IMU to shank misalignment error on 3-DOF knee angle estimation during different motions; (**a**–**c**), (**d**–**f**), (**g**–**i**) indicate the abduction, internal rotation and flexion angle of the knee during walking (3 km/h), jogging (6 km/h) and ordinary running (9 km/h), when added IMU to shank misalignment error from −10° to +10° in steps of 5°.

**Figure 7:**
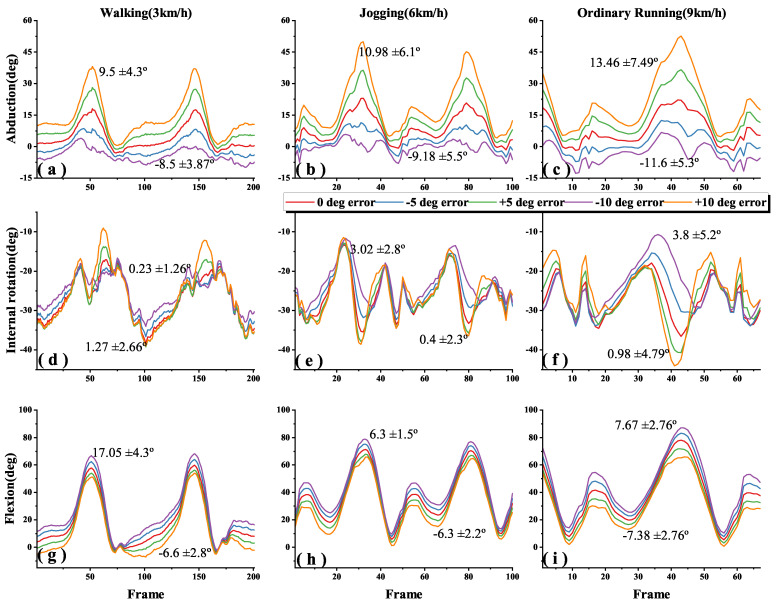
Effects of both IMU to thigh and shank misalignment error on 3-DOF knee angle estimation during different motions; (**a**–**c**), (**d**–**f**), (**g**–**i**) indicate the abduction, internal rotation and flexion angles of the knee during walking (3 km/h), jogging (6 km/h) and ordinary running (9 km/h), when added IMU to thigh and shank misalignment error from −10° to +10° in steps of 5°.

**Figure 8:**
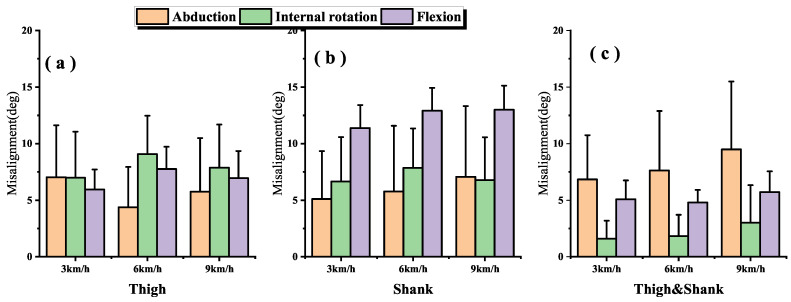
IMU to segment misalignment of all subjects. The bar and error bar represent the mean and standard deviation (SD) of the misalignment root mean square errors (RMSEs). (**a**–**c**) indicate the misalignment RMSEs of IMU to thigh misalignment, IMU to shank misalignment and IMU to thigh and shank misalignment, during different motions.

Figure 5, Figure 6 and Figure 7 shows the 3-DOF knee angle estimates after introducing errors to the I2S alignment parameters for the thigh, shank and both of them during walking, jogging and ordinary running. We have labeled the mean and standard deviation (SD) of the errors on the graph, where the lines clearly do not overlap. In order to make the image as clear as possible, we adjusted the X-axis for different speeds, but all statistical descriptive data such as mean, SD and root mean square errors (RMSEs) were calculated from the data of all experiments. Figure 8 shows the error results of the full experiments for all participants.

The dynamic I2S misalignment error has a small effect on the flexion angle and the error is stable close to a constant, which can be eliminated by subtracting the mean in the alignment phase [25]. Comparing the IMU-to-thigh misalignment error, the IMU-to-shank misalignment error had a greater effect on the estimation of the 3-DOF knee angle during walking and running, and this difference from the results in study [17] is due to the different types of motions being studied, and their I2S alignment parameters are obtained by static alignment. When the I2S misalignment error occurs both in the thigh and shank, as shown in Figure 8c, the estimation error of the 3-DOF knee angle is substantially reduced compared to the I2S misalignment error occurring alone. Though such a situation is unlikely to occur in real life, it can be studied as a control for the first two data sets.

#### 3.2.2. The 3-DOF Knee Angle Estimation Algorithm

**Figure 9:**
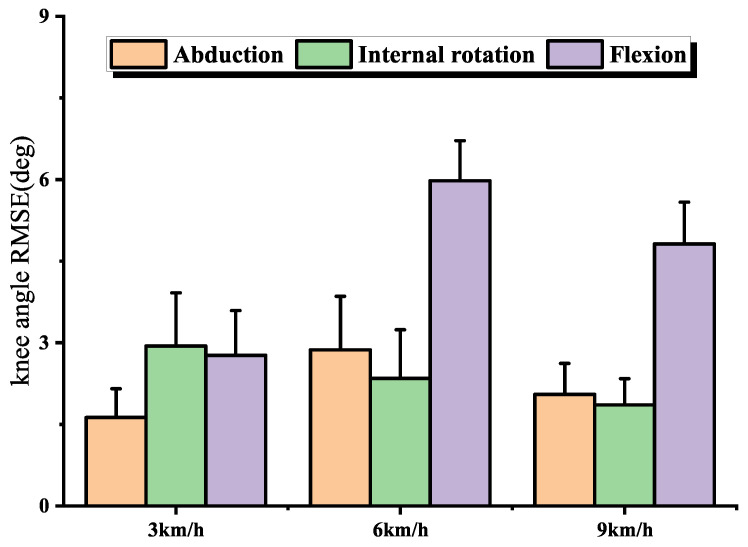
Comparison of IMU-based and optical motion capture system data-based 3-DOF knee angle estimations of RMSEs during different motions, the bar and error bar represent the mean and SD of the RMSEs.

We evaluated our knee angle estimation algorithm by taking the estimated knee angle based on IMU data as the observed value and the estimated knee angle based on the optical motion capture system as the reference. Figure 9 illustrates the RMSEs with no manual I2S misalignment error. Table 1 shows the maximum, minimum and range of motion (ROM) of the 3-DOF knee angle for all participants during walking, jogging and ordinary running.

During walking, the error in abduction angle is smaller compared to flexion angle and internal rotation angle, which is determined by the ROM of the knee in three dimensions during walking. When switching from 3 km/h walking to 6 km/h jogging, the relative values of abduction, flexion and internal rotation errors changed significantly, and when the running speed was accelerated, the relative values of errors did not change significantly, which indicates that the accuracy of IMU-based knee angle estimation during the switch from walking to running is noteworthy.

## 4. Discussion

### 4.1. Joint Constraint Optimization Algorithm

Under the combination of the above three particle swarm scales and the number of iterations (N = 60, D = 50; N = 60, D = 150; N = 160, D = 150), the groups with roughly the same positions of the first-iteration particles of the three algorithms are selected to draw images of particle fitness and iteration numbers (Figure 4). Result shows that our optimization algorithms all converge to near the optimization target before the 50th iteration, which indicates that the introduced algorithm has better search ability compared to the classical PSO and DPSO.

For the search problem in four-dimensional and six-dimensional space, unless the first-iteration particles randomly appear near the optimization target, the classical PSO algorithm is difficult to eventually converge to 0. After increasing the number of iterations and the particle swarm size, the classical PSO still could not converge to the optimization objective, which means that the classical PSO is not applicable in this study.

The introduction of dynamic inertia weight *w* greatly improves the convergence ability of particles (Figure 4 (blue lines)), but under the combination of three different iteration numbers and particle swarm size, the convergence is worse than that introduced by the new algorithm (Figure 4 (green lines)). The introduction of the crossover factor makes the convergence curve of our algorithm not smooth (Figure 4 (green lines)), the crossover between particles expands the search range, avoids falling into a local optimum and greatly reduces the number of iterations for particles to converge to the optimization target. In general, the introduced algorithm can satisfy the optimization of the objective function.

### 4.2. The Effects of Dynamic I2S Misalignment Error in Different Motions

Generally, in the IMU-based joint angle estimation, joint angle is defined by the rotation of the distal segment relative to the proximal segment. Figure 8a,b show that the knee angle estimation is more sensitive to IMU to shank misalignment error during walking and running motions, especially in flexion where the ROM is larger. For example, when adding a −10° error in the IMU to thigh alignment parameter, flexion accuracies across walking, jogging and ordinary running are 6.3 ± 2.2°, 8.96 ± 1.76° and 7.85 ± 3.4°, while adding a −10° error in the IMU to shank alignment parameter, are 17.1 ± 4.3°, 19.3 ± 2.9° and 19.2 ± 3.39° (Figure 5g–i and Figure 6g–i).

Since the error lines (Figure 5d–i and Figure 6d–i) of the internal rotation and flexion of the knee are partially parallel (i.e., constant errors). When the same misalignment error is added to both of the thigh and shank, the joint angle estimates are shifted in 3D space, making both the estimates of the internal rotation and flexion angles less sensitive to I2S misalignment than adding I2S errors to the thigh or shank alone (Figure 7d–i and Figure 8c).

- Abduction
  It is found in Figure 8a that the sensitivity of the abduction angle estimation to IMU to thigh misalignment error decreased when switching from walking to jogging; when speeding up from jogging to ordinary running, the sensitivity increased slightly, but still lower than walking. This is due to the fact that the human body stands for a shorter period in running than walking, which increases muscle activity and viscoelastic behavior of the soft tissues, and the increased muscle activity makes the abduction of the knee somewhat limited [21,26,27].
- Internal rotation
  Comparing Figure 8a,b, it is found that the effect of I2S misalignment error on the estimation of knee internal rotation becomes larger after switching from walking to running; while in the same motions, estimation of knee internal rotation is more sensitive to the IMU to thigh misalignment error than the IMU to shank misalignment error. This is due to the more significant internal rotation of the tibia during running than during walking [28]. During movements, the shank is affected by larger ground reaction forces, which makes the moment of the thigh greater and increases the internal rotation of the tibia [29], then the IMU to thigh misalignment error shows the greatest effect on the estimation of the internal rotation angle of the knee.
- Flexion
  It is found in Figure 8 that after introducing the I2S misalignment error, the mean value of flexion absolute error is larger but the SD is the smallest in the 3-DOF joint angle estimation. The large absolute error means are due to the fact that during walking and running, the knee joint moves mainly in the sagittal plane and the ROM of flexion is much greater than abduction and internal rotation [20,21,30]. However, the error brought by I2S misalignment error to flexion is mainly an approximate constant, which can be eliminated by subtracting the mean in the alignment phase [25], which explains why the effect brought by I2S misalignment error to flexion angle has the highest stability as well as the smallest SD. The IMU to shank misalignment error has the greatest effect on the estimation of the knee flexion angle, which is caused by the knee angle and is defined by the shank rotation relative to the thigh.

### 4.3. Joint Angle Estimation

A comparison of the IMU-based and optical motion capture system-based full-process 3-DOF knee angle estimates is show in Figure 9. The RMSEs ranged from 1.6° to 5.9° during all trials. In the same walking scenario, the RMSEs of our 3-DOF knee angle estimation algorithm ranged from 1.6° to 2.9°, which was better than the 2.3° to 5.6° of the study [23]. It indicates that our 3-DOF knee angle estimation algorithm shows good accuracy.

In order to study the correlation between the error data of the three groups of walking, jogging and ordinary running, the data were first tested for normality at *p* = 0.05, and some of the data samples did not meet the assumption of normal distribution, so the Friedman ANOVA multiple independent non-parametric test was used to analyze the differences between the three groups of data.

When the human body switched from walking to ordinary running, the estimation errors of both abduction and flexion angles did not increase in a positive proportion with increasing speed. The results showed significant differences (p<0.01) in the RMSEs of knee angle estimation during walking and running, and no significant differences (p>0.05) in the RMSEs of knee angle estimation during running at different speeds, suggesting that the differences in joint kinematics during the switch from walking to running in humans is worth investigating.

## 5. Conclusions

In this paper, based on the joint constraint model, we introduced the DPSO algorithm with crossover factor to obtain the dynamic I2S alignment parameters, the effectiveness of the introduced optimization algorithm is experimentally demonstrated. Using the dynamic I2S alignment parameters, we performed the estimation algorithm of 3-DOF joint angles. A comparison of the results of IMU-based and optical motion capture system-based joint angle estimation throughout the process. The range of 3-DOF knee angle RMSEs is 1.6°–5.9° during different motions. Significant differences in RMSEs are found between walking and running, which also demonstrated significant kinematic differences in the knee joint during walking and running.

Experiments are designed in which human walking (3 km/h), jogging (6 km/h) and ordinary running (9 km/h) were used to investigate the effects of dynamic I2S misalignment error on the estimation of 3-DOF knee angle during different motions by manually adding errors to the I2S alignment parameter.

Through experiments, we found that the effect of IMU to thigh misalignment error on knee abduction angle estimation decreases when switching from walking to running, when speeding up from jogging to ordinary running, the effects become larger.

When switching from walking to running, the effect of I2S misalignment error on the estimation of knee internal rotation angle becomes larger, and the effect of IMU to thigh misalignment error on the estimation of knee internal rotation angle is always larger than IMU to shank misalignment error regardless of the motion.

The effect of IMU to shank misalignment error on knee flexion angle estimation is numerically larger than the other two degrees of freedom, but among all motions, the effect of I2S misalignment on knee flexion angle estimation is close to constant, and the SD of the error is smaller than the other two degrees of freedom.

A limitation of this study is that we only experimentally confirmed and quantified the difference in the effect of I2S misalignment error on 3-DOF joint angle estimation during different motions, but a specific method for error elimination was not proposed. In the future, we should build corresponding models for the effect of different I2S misalignment errors on 3-DOF joint angle estimation to eliminate the discretization errors in order to obtain more accurate evaluation of joint angles.

## Figures and Tables

**Table 1:** This table shows the maximum, minimum and range of motion (ROM) values of the estimated 3-DOF knee angle during walking, jogging and ordinary running for all participants.

Motions	3-DOF Knee Angle	Max (°)	Min (°)	ROM (°)
Walking (3 km/h)	abduction	18.08898	−3.00719	21.09617
	internal rotation	−17.0804	−38.3691	21.28875
	flexion	59.85131	−3.28834	63.13965
Jogging (6 km/h)	abduction	23.32207	−1.75181	25.07388
	internal rotation	−13.3855	−35.9901	22.60462
	flexion	72.80989	5.867394	66.9425
Ordinary Running (9 km/h)	abduction	22.24302	−2.22884	24.47186
	internal rotation	−17.9308	−37.9682	20.03737
	flexion	80.15724	5.39035	74.76689

## Data Availability

All measurement data in this paper has been listed in the content of the article, which can be used by all peers for related research.
